# Modulation of NFκB Signaling by Natural Compounds in Sarcoma and Normal Muscle Models

**DOI:** 10.3390/ijms27115025

**Published:** 2026-06-02

**Authors:** Justyna Radzka, Agnieszka Gizak, Dagmara Baczyńska, Adam Junka, Bartłomiej Dudek, Malwina Brożyna, Anna Szewczyk, Julita Kulbacka

**Affiliations:** 1 Department of Molecular Physiology and Neurobiology, Faculty of Biology, University of Wroclaw, 50-137 Wroclaw, Poland; justyna.radzka@uwr.edu.pl (J.R.); agnieszka.gizak@uwr.edu.pl (A.G.); 2 Department of Molecular and Cellular Biology, Faculty of Pharmacy, Wroclaw Medical University, Borowska 213, 50-556 Wroclaw, Poland; dagmara.baczynska@umw.edu.pl (D.B.); a.szewczyk@umw.edu.pl (A.S.); 3 “P.U.M.A.” Platform for Unique Model Application, Department of Translative Technologies, Faculty of Pharmacy, Wroclaw Medical University, Borowska 213, 50-556 Wroclaw, Poland; adam.junka@umw.edu.pl (A.J.); bartlomiej.dudek@umw.edu.pl (B.D.); malwina.brozyna@umw.edu.pl (M.B.); 4 College of Life Sciences and Medicine, Zhejiang Sci-Tech University, Hangzhou 310018, China; 5 Department of Immunology and Bioelectrochemistry, State Research Institute Centre for Innovative Medicine, Santariškių 5, 08410 Vilnius, Lithuania

**Keywords:** muscle cancer, berberine, curcumin, cucurbitacin E (CurE), biochanin A, caffeic acid phenethyl ester (CAPE)

## Abstract

Berberine, curcumin, biochanin A, cucurbitacin E, and caffeic acid phenethyl ester (CAPE) are plant-derived compounds with long histories of use in traditional medicine for inflammatory and proliferative conditions. Their known capacity to modulate NF-κB signaling makes them candidates for anticancer investigation, particularly in mesenchymal malignancies such as fibrosarcoma, which arise in muscle-rich environments shared with normal myogenic tissue. To evaluate the selective anticancer potential of these compounds in fibrosarcoma (WEHI-164) and normal muscle (L6) cells, with focus on mitochondrial function, mitophagy, cellular senescence, and NF-κB-related metabolic pathways, alongside preliminary in vivo toxicity assessment. IC_50_ values were determined using MTT and PrestoBlue^®^ assays. Mitochondrial membrane potential was assessed using JC-1 and normalized to the matched untreated control for each cell line, and mitophagy by PINK1/PARKIN immunofluorescence colocalisation together with a mitophagy dye assay. Cellular senescence was measured using a β-galactosidase assay, and ATP levels by a luminescence-based method. Gene expression of NF-κB pathway components and PFKFB3 was analyzed by RT-qPCR. In vivo–like toxicity was assessed using the *Galleria mellonella* model, including PBS handling, DMSO vehicle, and 70% ethanol utility controls, with survival data analyzed by Kaplan–Meier curves and the log-rank test. The compounds differentially affected normal and cancer cells, indicating selectivity toward malignant phenotypes. Decreased ATP and mitochondrial depolarization suggest disruption of bioenergetic homeostasis, supported by modulation of mitophagy. Stronger effects in WEHI-164 cells indicate higher susceptibility to mitochondrial dysfunction. Increased cellular senescence suggests inhibition of tumor proliferation. These findings indicate that natural NF-κB modulators may exert anticancer effects by targeting mitochondrial and metabolic homeostasis. Differential sensitivity between normal and tumor cells highlights therapeutic potential. In the *G. mellonella* model, berberine and curcumin did not differ significantly from the PBS or DMSO controls, whereas CAPE, CurE, and particularly biochanin A produced significantly greater larval mortality. The *G. mellonella* assay should be regarded only as a preliminary acute toxicity screen, and further in vivo studies in mammalian models are required to clarify mechanisms and clinical relevance.

## 1. Introduction

Sarcomas comprise a diverse group of malignant tumors of mesenchymal origin that arise in soft tissues or bone and frequently develop in anatomical sites rich in skeletal muscle and connective tissue. Despite major advances in molecular diagnostics and subtype stratification, sarcomas remain clinically challenging because of their biological heterogeneity, variable therapeutic vulnerabilities, and a persistent risk of recurrence or metastatic spread [1,2]. Soft-tissue sarcomas encompass more than 100 histological entities with distinct molecular drivers, highlighting the need for mechanism-informed therapeutic approaches [1,2]. Because many sarcomas develop within or adjacent to skeletal muscle, therapeutic strategies must balance anticancer efficacy with preservation of muscle viability, mitochondrial function, and regenerative capacity.

A growing body of evidence indicates that sarcoma genesis and progression are shaped by inflammatory and stress-related signaling operating both within tumor cells and in the tumor microenvironment [3]. Among the pathways involved, the nuclear factor kappa-light-chain-enhancer of activated B cells (NF-κB) occupies a central position. NF-κB integrates extracellular and intracellular stress signals, including cytokines and reactive oxygen species (ROS), to regulate transcriptional programs controlling inflammation, cell survival, metabolism, and senescence [4,5,6,7]. In cancer, NF-κB can promote tumor cell survival, metabolic adaptation, and resistance to stress, making it an attractive, although complex, therapeutic target [4,5,6,7,8,9,10].

The NF-κB family comprises five Rel proteins—RelA (p65), RelB, c-Rel, NF-κB1 (p50/p105), and NF-κB2 (p52/p100), which form transcriptionally active dimers regulated through canonical and non-canonical pathways [7,11,12,13,14]. NF-κB signaling outcomes are strongly cell-type- and context-dependent, and its activity in tumor cells may differ substantially from NF-κB programs in normal tissues such as skeletal muscle [9,15].

Sarcoma cells can engage NF-κB-dependent transcriptional programs that support metabolic remodeling and stress adaptation [3,4,16]. At the same time, NF-κB plays essential roles in skeletal muscle homeostasis, influencing myogenesis, regeneration, and inflammatory responses [17,18,19,20]. Persistent NF-κB activation in muscle can impair differentiation and contribute to muscle dysfunction, highlighting its dual relevance in tumor biology and normal tissue physiology. These considerations motivate comparative approaches that evaluate NF-κB modulation in both tumor and muscle cells.

Natural compounds have long provided leads for anticancer drug discovery, and many phytochemicals exhibit anti-inflammatory and anticancer activities linked to modulation of NF-κB signaling and related stress-response pathways [21,22,23,24,25]. Unlike highly selective inhibitors, natural compounds often exert multi-target effects, influencing inflammatory signaling, mitochondrial function, and cellular metabolism. Recent reports continue to expand this evidence base: plant-derived extracts and phytochemical formulations have been shown to modulate inflammatory and proliferative pathways in both skin-barrier and breast cancer models, supporting the ongoing relevance of natural compounds as NF-κB-associated bioactives [24,25]. However, this pleiotropy raises the possibility of toxicity in normal cells, underscoring the need for comparative evaluation.

The present manuscript focuses on natural compounds previously associated with NF-κB modulation: curcumin, berberine, caffeic acid phenethyl ester (CAPE), biochanin A, and cucurbitacin E (CurE). Curcumin, a polyphenol derived from Curcuma longa, is a well-known anti-inflammatory and anticancer agent that inhibits NF-κB activation, although its clinical application is limited by poor bioavailability [26,27]. CAPE, a component of propolis, has been widely used as an inhibitor of NF-κB-dependent signaling [28]. Biochanin A, an isoflavone present in plants such as red clover, exhibits anti-inflammatory and anti-proliferative activity associated with inhibition of NF-κB signaling [29]. Berberine, an isoquinoline alkaloid, suppresses inflammatory responses via AMPK activation and can also affect mitochondrial function, including inhibition of respiratory complex I [30,31]. Cucurbitacin E, a tetracyclic triterpenoid, has been reported to inhibit NF-κB-associated pathways and inflammatory signaling in experimental models [32].

NF-κB signaling is closely linked to mitochondrial function and cellular metabolism.

Mitochondria regulate ATP production, redox balance, and stress signaling, and inflammatory signaling reciprocally remodels mitochondrial morphology and respiratory capacity, with consequences for both cancer cells and myogenic cells [33]. In skeletal muscle, pro-inflammatory NF-κB signaling has been associated with mitochondrial disturbances and insulin signaling defects under metabolic stress [33]. In tumors, mitochondrial rewiring contributes to biosynthetic demands and stress tolerance, and NF-κB-related pathways can support these adaptations by regulating metabolic enzymes and transporters [4,5,6,7,16]. Mitophagy, particularly the PINK1/PARKIN pathway, represents an important mitochondrial quality-control mechanism activated in response to mitochondrial depolarization. Loss of mitochondrial membrane potential promotes PINK1 accumulation on the outer mitochondrial membrane, recruitment and activation of the E3 ligase PARKIN, ubiquitination of mitochondrial substrates, and subsequent autophagic clearance [34]. Because many NF-κB modulators influence redox balance, mitochondrial integrity, or autophagy-associated signaling, mitophagy represents a biologically informative endpoint for comparing tumor and normal cell responses. Cellular senescence is another stress-associated phenotype regulated, in part, by NF-κB. Senescence involves durable proliferative arrest and inflammatory signaling, and can contribute to tumor suppression or therapy resistance depending on context [35,36,37]. In skeletal muscle, senescence-like states may impair regenerative capacity, highlighting the importance of evaluating senescence alongside mitochondrial and viability-related endpoints.

Finally, glycolytic regulation provides an additional perspective on NF-κB-associated metabolic adaptation. The glycolytic activator PFKFB3 is frequently implicated in cancer metabolism by promoting glycolytic flux, and its regulation may reflect shifts in bioenergetic programs associated with NF-κB signaling. Evaluating PFKFB3 together with NF-κB subunits therefore helps determine whether NF-κB modulation is accompanied by metabolic reprogramming, and tumor–myogenic comparisons remain essential to establish whether such modulation can be achieved with tumor selectivity while preserving normal muscle function.

Accordingly, the present study was designed to compare the effects of curcumin, berberine, CAPE, biochanin A, and CurE in a murine fibrosarcoma model (WEHI-164) and a normal myogenic model (L6). The study evaluates cell viability, mitochondrial membrane potential, ATP-related bioenergetics, mitophagy-related PINK1/PARKIN signals, cellular senescence, and transcriptional changes in NF-κB pathway components and the metabolic regulator PFKFB3. In addition, a *Galleria mellonella* larval assay was used as an in vivo-like screening platform to provide an initial indication of organismal toxicity.

By comparing tumor and normal muscle models across NF-κB-associated cellular responses, this study examines whether natural NF-κB modulators preferentially affect sarcoma cells or produce similar stress-related effects in normal myogenic cells.

## 2. Results

### 2.1. Cytotoxicity of the Tested Compounds and Determination of the IC_50_ Parameter

**Figure 1:**
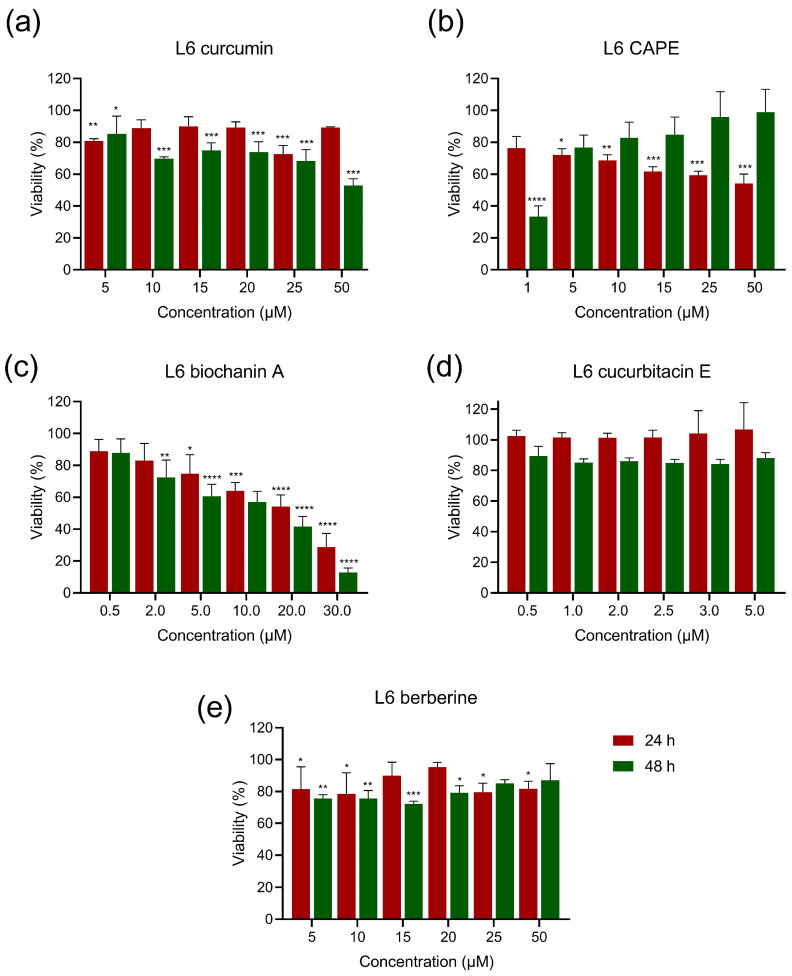
Viability of the L6 cells measured by the MTT assay after 24 and 48 h of treatment with curcumin (**a**), CAPE (**b**), biochanin A (**c**), cucurbitacin E (**d**), and berberine (**e**). Error bars represent ± SD for N = 3. Statistical significance relative to the control cells of the respective line was determined using one-way analysis of variance (ANOVA), where * indicates *p* < 0.05, ** *p* < 0.001, and *** *p* < 0.005, **** *p* < 0.001.

The MTT assay was used to evaluate the cytotoxic effects of biochanin A, CAPE, CurE, curcumin, and berberine. Figure 1 presents the response of L6 cells after 24 h and 48 h incubation with the tested compounds. Treatment with berberine (Figure 1e) and CurE (Figure 1d) did not result in significant changes in mitochondrial activity in L6 cells compared with the control group. In contrast, biochanin A (Figure 1c) induced a concentration-dependent cytotoxic effect at both time points. At the highest concentration (30 μM), cell viability decreased to approximately 30% after 24 h and further declined to about 18% after 48 h. Treatment with curcumin (Figure 1a) also resulted in a pronounced cytotoxic effect, particularly after prolonged exposure (48 h). In the case of CAPE (Figure 1b), the strongest cytotoxic effect was observed at 1 μM after 48 h. Interestingly, at higher CAPE concentrations and after extended incubation (48 h), cell viability increased. This effect may be associated with intracellular degradation or reduced CAPE stability.

**Figure 2:**
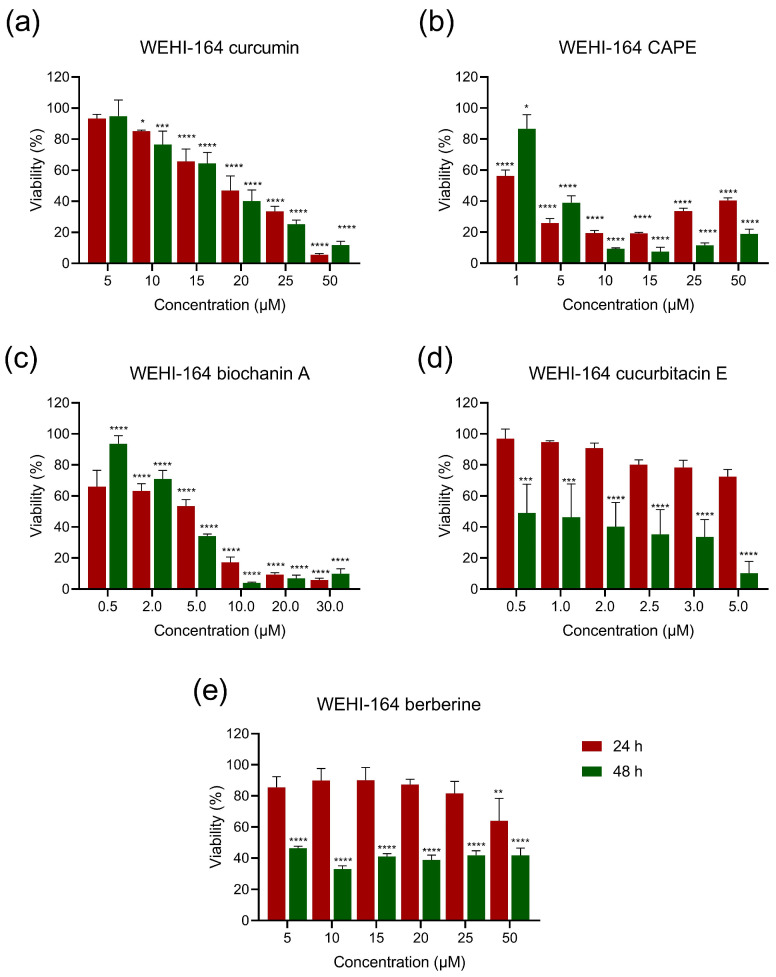
Viability of the WEHI-164 cells measured by the MTT assay after 24 and 48 h of treatment with curcumin (**a**), CAPE (**b**), biochanin A (**c**), cucurbitacin E (**d**), and berberine (**e**). Error bars represent ± SD for N = 3. Statistical significance relative to the control cells of the respective line was determined using one-way analysis of variance (ANOVA), where * indicates *p* < 0.05, ** *p* < 0.001, *** *p* < 0.005, and **** *p* < 0.001.

Figure 2 presents the response of WEHI-164 cells after 24 h and 48 h treatment with the tested compounds. WEHI-164 cells exhibited increased sensitivity to all tested compounds, compared with untreated cells. In the case of berberine (Figure 2e), the strongest cytotoxic effect was observed after 48 h of incubation at 10 μM, with cell viability reduced to approximately 33%. A pronounced decrease in viability was observed in cells incubated with biochanin A (Figure 2c) at 10–30 μM, with viability decreasing to approximately 17–9% after 24 h. During incubation with CurE (Figure 2d) and curcumin (Figure 2a), cell viability significantly decreased with increasing concentration and incubation time. All analyzed compounds exhibited stronger cytotoxic effects on WEHI-164 tumor cells than on normal L6 myoblast cells.

**Figure 3:**
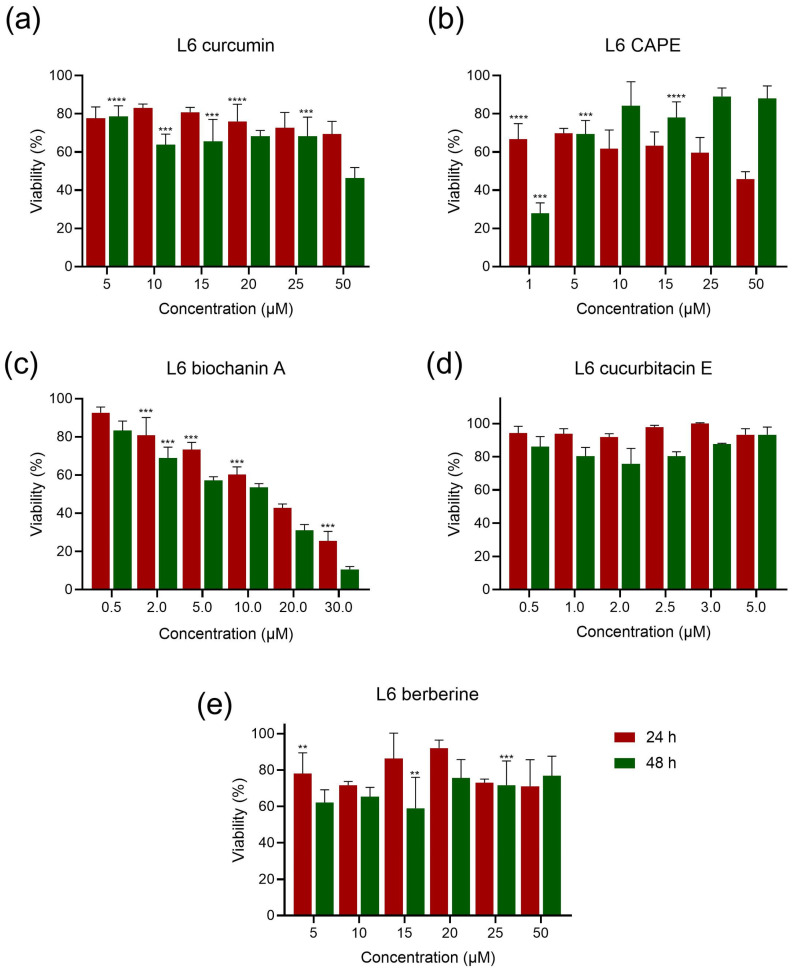
Viability of the L6 cells measured by the PrestoBlue^®^ assay after 24 and 48 h of treatment with curcumin (**a**), CAPE (**b**), biochanin A (**c**), cucurbitacin E (**d**), and berberine (**e**). Error bars represent ± SD for N = 3. Statistical significance relative to the control cells of the respective line was determined using one-way analysis of variance (ANOVA), where ** indicates *p* < 0.001, *** *p* < 0.005, and **** *p* < 0.001.

An additional PrestoBlue^®^ assay was performed to assess cell viability after 24 h and 48 h treatment of L6 and WEHI-164 cells with the tested compounds. After treatment with curcumin (Figure 3a), a significant decrease in cell viability was observed at 10 µM and above, at both 24 and 48 h of exposure. At the highest concentration tested (50 µM), viability decreased to approximately 60% of the control. In L6 cells treated with CAPE (Figure 3b), the cytotoxic effect was most pronounced at the lowest concentration (1 µM), where cell viability dropped below 40%. At higher concentrations, this effect was less evident, and viability remained between 60% and 80%. Biochanin A exhibited a clear concentration-dependent effect, with a gradual decline in cell viability, beginning at 2 µM and reaching approximately 20% at 30 µM (Figure 3c). Treatment with CurE did not result in significant changes in L6 cell viability compared with the control group (Figure 3d). Berberine caused a moderate reduction in cell viability, particularly at concentrations of 10–25 µM, where viability decreased to approximately 60–70% (Figure 3e). This effect was slightly more pronounced after 48 h of treatment.

**Figure 4:**
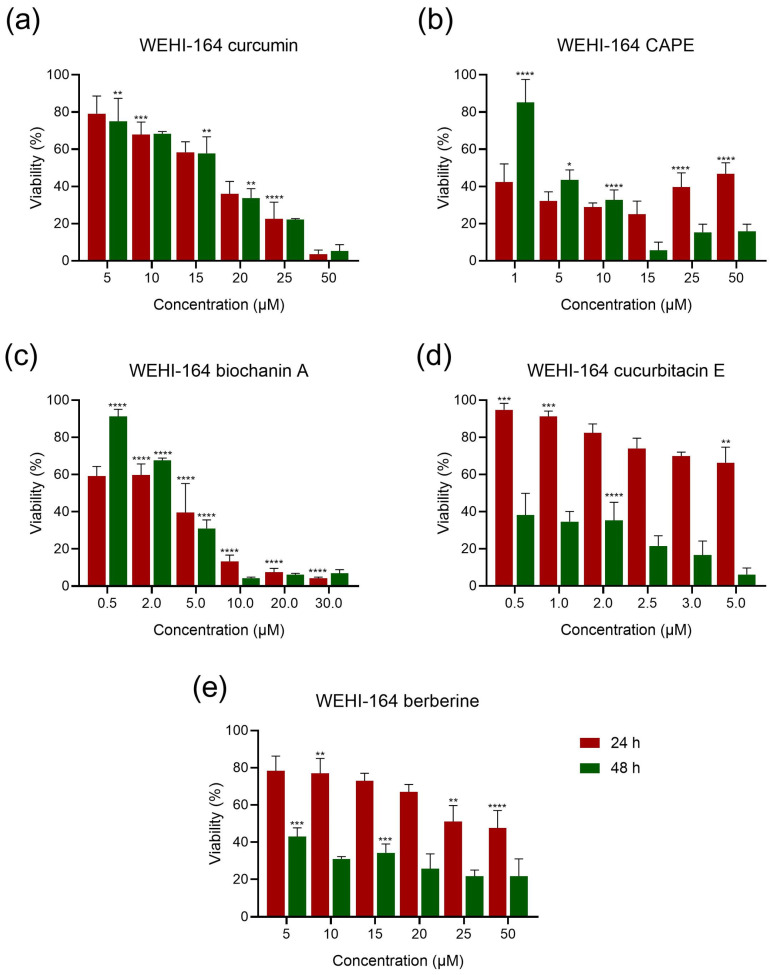
Viability of the WEHI-164 cell line measured by the PrestoBlue^®^ assay after 24 and 48 h of treatment with curcumin (**a**), CAPE (**b**), biochanin A (**c**), cucurbitacin E (**d**), and berberine (**e**). Error bars represent ± SD for N = 3. Statistical significance relative to the control cells of the respective line was determined using one-way analysis of variance (ANOVA), where * indicates *p* < 0.05, ** *p* < 0.001, *** *p* < 0.005, and **** *p* < 0.001.

After incubation of WEHI-164 cells with curcumin, a clear, concentration-dependent decrease in cell viability was observed. A significant effect was evident at 10 µM, and at 25–50 µM, cell viability dropped below 20% of the control (Figure 4a). The effect was more pronounced after 48 h of incubation. CAPE markedly reduced the viability of cancer cells across a wide range of concentrations. At 5–10 µM, viability decreased to approximately 30–40%, and at higher concentrations, it remained at a similar level (Figure 4b). The effect intensified with prolonged incubation time. Biochanin A exhibited a very strong, dose-dependent cytotoxic effect. A significant reduction in viability was observed starting from 2 µM, and at 20–30 µM, cell viability was nearly abolished (Figure 4c). WEHI-164 cells treated with CurE showed a moderate decrease in viability at 1–5 µM, particularly after 48 h of treatment, when viability dropped to 20–40% (Figure 4d). Berberine demonstrated a clear, dose-dependent cytotoxic effect. A reduction in cell viability to below 50% of control was observed starting at 10 µM, and at 50 µM, viability did not exceed 20% (Figure 4e).

Table 1 and Table 2 below summarize IC50 values after 24 h (Table 1) and 48 h (Table 2) exposure for five natural compounds in normal L6 muscle cells and WEHI 164 sarcoma cells. The obtained results indicated that curcumin and CAPE exhibited preferential cytotoxicity toward the sarcoma cell line, whereas biochanin A, berberine, and especially CurE were relatively more toxic to normal L6 cells.

### 2.2. Assessment of Mitochondrial Polarization in L6 and WEHI-164 Cells Following Exposure to Cytotoxic Compounds

**Figure 5:**
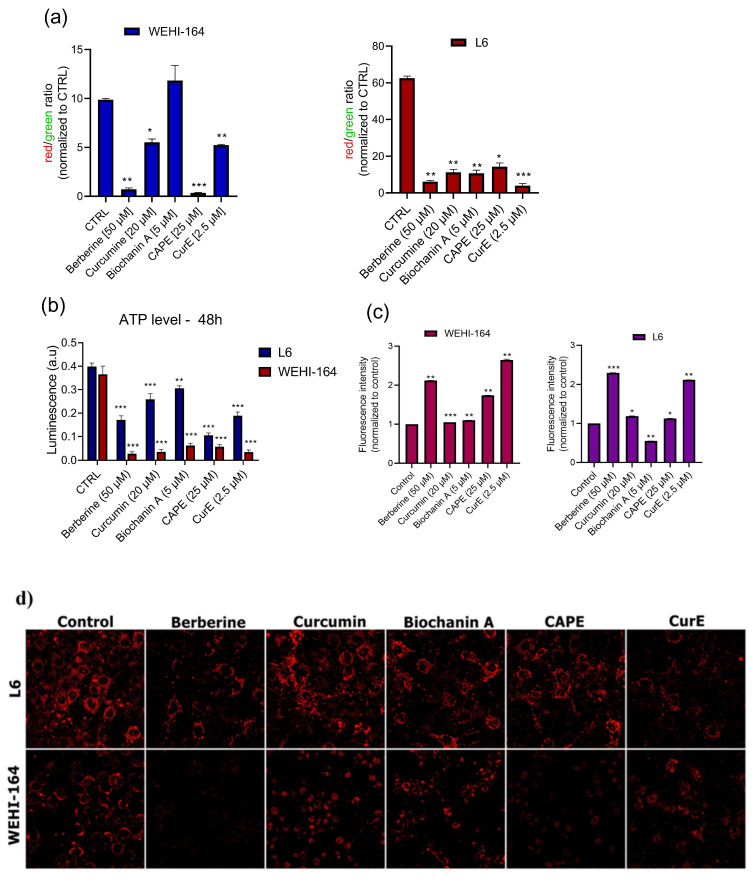
Effects of berberine (50 μM), curcumin (20 μM), biochanin A (5 μM), CAPE (25 μM), and CurE (2.5 μM) on L6 and WEHI-164 cells. (**a**) Mitochondrial membrane polarization after 24 h exposure, expressed as JC-1 red/green fluorescence ratio normalized to the matched untreated control of each cell line. (**b**) Intracellular ATP levels after 48 h exposure, expressed in arbitrary luminescence units. (**c**) Mitophagy intensity after 24 h exposure, expressed as fluorescence intensity normalized to the matched untreated control of each cell line. (**d**) Representative CLSM images of L6 and WEHI-164 cells stained with JC-1 after 24 h incubation with the tested compounds; scale bar—20 μm. Data are presented as mean ± SD. Statistical significance relative to the matched untreated control for each cell line was assessed using one-way ANOVA: * *p* < 0.05; ** *p* < 0.01; *** *p* < 0.001. Images taken using CLSM. Scale bar 20 μm.

Mitochondrial membrane polarization was assessed in L6 and WEHI-164 cell lines using the JC-1 dye. The results were expressed as the ratio of red to green fluorescence, normalized to the control. In L6 myoblasts, all tested compounds induced a pronounced reduction in mitochondrial membrane potential, as evidenced by a substantial decrease in the JC-1 red/green fluorescence ratio (Figure 5a). Given the high basal mitochondrial polarization observed in control L6 cells (mean ≈ 62.55), the reduction to values ranging from 3.98 to 14.31 indicates marked mitochondrial dysfunction. The strongest depolarizing effect was observed for CurE (mean ≈ 3.98; ~94% reduction) and berberine (mean ≈ 6.13; ~90% reduction), suggesting a profound disruption of mitochondrial integrity. Curcumin and biochanin A reduced the membrane potential by approximately 82–83%, whereas CAPE induced a slightly less pronounced, though still substantial, decrease (~77%).

In contrast to L6 cells, WEHI-164 fibrosarcoma cells demonstrated a more heterogeneous response (Figure 5a). The basal mitochondrial membrane potential in control cells was markedly lower (mean ≈ 9.87), reflecting intrinsic metabolic differences between tumor-derived and non-transformed cells. Berberine (mean ≈ 0.73; ~93% reduction) and CAPE (mean ≈ 0.36; ~96% reduction) induced profound mitochondrial depolarization, indicating strong destabilization of mitochondria. These effects were proportionally comparable to those observed in L6 cells, despite the lower baseline values. Curcumin and CurE produced moderate depolarization (reductions of approximately 44–47%), suggesting partial impairment of mitochondrial function rather than complete collapse of membrane potential. Notably, biochanin A exerted a distinct effect in this cell line. Instead of reducing mitochondrial polarization, it increased the red/green ratio to approximately 11.83 (around 20% above control), indicating preservation or enhancement of mitochondrial membrane potential. This differential response suggests that biochanin A does not trigger mitochondrial dysfunction in WEHI-164 cells and may even exert a stabilizing effect on mitochondrial integrity in this tumor model.

A comparison of responses between the cell lines showed that WEHI-164 cells were more sensitive to berberine and CAPE than L6 cells, showing almost complete depolarization with these substances. In contrast, in the L6 line, CurE and berberine induced the strongest membrane depolarization, while the other compounds caused moderate changes. Biochanin A exerted the weakest effect on mitochondrial membrane polarization in both cell lines, although the effect was more pronounced in L6 cells than in WEHI-164 cells. These findings indicate differential mitochondrial sensitivity between normal muscle and cancer cells in response to the tested natural compounds.

### 2.3. Changes in ATP Levels in the Investigated Cell Lines

Intracellular ATP levels were assessed following 48 h of exposure of L6 and WEHI-164 cells to selected NF-κB inhibitors. Luminescence intensity, reflecting cellular metabolic activity, was used as an indicator of treatment-induced cytotoxicity (Figure 5b).

In L6 myoblasts, the mean ATP level in control cells was 0.398 ± 0.016 A.U. Following 48 h of exposure, all tested compounds reduced intracellular ATP levels compared with the control. Berberine (50 μM) decreased ATP levels by approximately 57% compared with the control. Curcumin (20 μM) resulted in a reduction of approximately 35%. Biochanin A (5 μM) exerted a comparatively moderate effect, lowering ATP levels by approximately 23%. More pronounced effects were observed following treatment with CAPE (25 μM) and CurE (2.5 μM). CAPE reduced ATP levels to 0.106 ± 0.010 A.U., corresponding to a decrease of approximately 73%, whereas CurE decreased ATP to 0.189 ± 0.017 A.U. (approximately 52% reduction). In summary, the strongest inhibition of ATP production in L6 cells was observed for CAPE and berberine, while biochanin A demonstrated the weakest effect.

In WEHI-164 fibrosarcoma cells, the mean ATP level in untreated control cells was 0.365 ± 0.034 A.U. All tested compounds induced a markedly greater reduction in ATP levels compared with that observed in L6 cells. Berberine (50 μM) reduced ATP levels by approximately 92% relative to control. Curcumin (20 μM) and CurE (2.5 μM) each resulted in an approximately 90% reduction in ATP levels. Biochanin A (5 μM) and CAPE (25 μM) also significantly decreased ATP levels, corresponding to reductions of approximately 83% and 84%, respectively. Thus, in WEHI-164 cells, all tested compounds induced profound depletion of intracellular ATP, with berberine producing the most pronounced effect.

Although basal ATP levels were comparable in untreated L6 (0.398 ± 0.016 A.U.) and WEHI-164 cells (0.365 ± 0.034 A.U.), treatment-induced ATP depletion was consistently more pronounced in WEHI-164 cells. Reductions ranged from 83 to 92% in WEHI-164 cells compared with 23–73% in L6 cells, with biochanin A showing the greatest differential effect (23% vs. 83%), whereas CAPE produced similar reductions in both cell lines (73% vs. 84%). Overall, WEHI-164 cells exhibited substantially greater sensitivity to all tested compounds, indicating tumor-preferential cytotoxic activity.

### 2.4. The Effect of Selected Cytotoxic Compounds on Mitophagy in L6 and WEHI-164 Cell Lines

After 24 h of treatment with the tested compounds, differential effects on mitophagy levels were observed in L6 cells. Mitophagy was quantified as fluorescence intensity normalized to the control (set to 1). Treatment with berberine (50 μM) resulted in a pronounced, approximately 2.3-fold (***) increase in mitophagy. Similarly, CurE (2.5 μM) markedly, 2.1-fold (**), enhanced mitophagy relative to control. More moderate but statistically significant elevations were observed following treatment with curcumin (20 μM) and CAPE (25 μM). Curcumin increased fluorescence almost 1.2-fold, while CAPE resulted in approximately 15% increase over control (*). In contrast, biochanin A (5 μM) significantly reduced mitophagy, as indicated by fluorescence intensities representing a reduction of approximately 45% compared with untreated cells (**). Collectively, these data indicate that in L6 myoblasts, berberine and CurE are potent inducers of mitophagy, curcumin and CAPE exert moderate stimulatory effects, whereas biochanin A significantly suppresses mitochondrial turnover (Figure 5c).

Mitophagy in WEHI-164 fibrosarcoma cells was expressed as fluorescence intensity normalized to the control (1). Treatment with berberine (50 μM) resulted in a significant, approximately 2.1-fold, increased mitophagy relative to control (**). The strongest induction of mitophagy was observed following CurE (2.5 μM) treatment, corresponding to approximately a 2.6-fold increase compared with untreated cells (**). A pronounced stimulatory effect was also detected for CAPE (25 μM), which increased mitophagy over 1.7-fold (**) (74% elevation over control). In contrast, curcumin (20 μM) produced only a modest increase, reflecting a 5% elevation (*), while biochanin A (5 μM) resulted in a similarly mild increase, corresponding to values approximately 10% above control. Overall, all tested compounds enhanced mitophagy in WEHI-164 cells, although the magnitude of the response varied substantially, with CurE exerting the most pronounced effect (Figure 5c).

Both berberine and CurE markedly stimulated mitophagy in L6 and WEHI-164 cells. However, while berberine induced a comparable increase in both lines (≈2.30-fold in L6 vs. ≈2.12-fold in WEHI-164), CurE exerted a substantially stronger effect in WEHI-164 cells (≈2.6-fold) than in L6 cells (≈2.1-fold), indicating a somewhat heightened sensitivity of tumor cells to this compound. A slightly higher divergence was observed for CAPE. In L6 cells, CAPE produced only a modest increase (≈1.1-fold), whereas in WEHI-164 cells it induced a robust enhancement (≈1.7-fold), suggesting a moderately tumor-selective potentiation of mitophagy. On the other hand, biochanin A exerted opposite effects in the two models. In L6 myoblasts, it significantly reduced mitophagy to approximately 45% of the control level, whereas in WEHI-164 cells, it slightly increased mitochondrial turnover to about 110% of the control level. This differential response suggests cell-type-specific modulation of mitochondrial quality control mechanisms. Finally, curcumin induced a moderate (≈1.2-fold) increase in L6 cells, but only a minimal (≈1.05-fold) elevation in WEHI-164 cells, indicating a stronger responsiveness of non-transformed myoblasts to this compound. Taken together, these findings demonstrate that although both cell lines respond to the tested natural compounds with alterations in mitophagy, tumor-derived WEHI-164 cells exhibit a markedly stronger induction in response to CurE and CAPE, whereas biochanin A selectively suppresses mitophagy in normal L6 myoblasts. These differences suggest cell-type-specific regulation of mitochondrial turnover and may reflect distinct metabolic vulnerabilities between normal and cancerous muscle-derived cells.

### 2.5. Expression and Localization of PINK-1 and PARKIN in L6 and WEHI-164 Cells After Exposure to Cytotoxic Compounds

**Figure 6:**
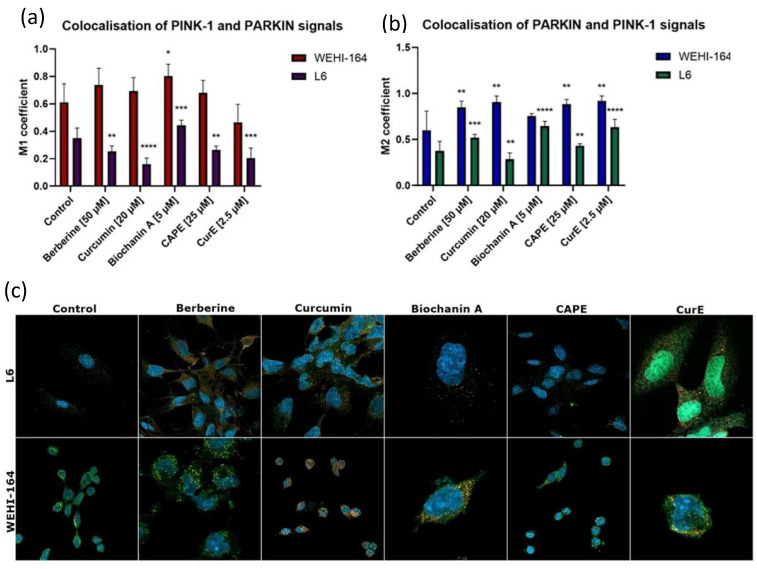
M1 coefficient values, defining the percentage of PINK-1 fluorescent signal overlapping with PARKIN signal, in WEHI-164 and L6 cells after 24 h of exposure to the tested compounds. Error bars are ± SD for N = 7. Statistical significance compared to control cells of a given line was determined by one-way analysis of variance (ANOVA), where * is statistically significant for *p* < 0.05, ** for *p* < 0.001, *** *p* < 0.005, and **** *p* < 0.001 (**a**). M2 coefficient values, which determine the percentage of PARKIN fluorescent signal colocalising with PINK-1 signal, in WEHI-164 and L6 cells after 24 h exposure to the tested compounds. Error bars are ± SD for N = 7. Statistical significance compared to control cells of a given line was determined by one-way analysis of variance (ANOVA), where ** statistically significant for *p* < 0.001, *** *p* < 0.005, and **** *p* < 0.001 (**b**). Presentation of the effect of berberine (50 μM), curcumin (20 μM), biochanin A (5 μM), CAPE (25 μM), and CurE (2.5 μM) on L6 and WEHI-164 cells after 24 h of incubation. Nuclei are stained blue with DAPI, PARKIN is stained red with AlexaFluor 647 and PINK-1 is stained green with AlexaFluor 647. Images taken using CLSM. Scale bar—20 μm (**c**).

Manders’ overlap coefficient was used to quantify the colocalization between PINK-1 and PARKIN immunofluorescence signals in WEHI-164 and L6 cells following 24 h exposure to the tested compounds. In control cells of both lines, the M1 coefficient indicated a basal level of colocalization between PINK-1 and PARKIN (Figure 6a); however, this basal interaction was markedly higher in WEHI-164 cells than in L6 myoblasts.

In WEHI-164 cells, the control group exhibited a mean M1 value of 0.628 ± 0.146, indicating a relatively high basal proportion of total PINK-1 signal colocalizing with PARKIN. Treatment with berberine (50 μM) increased the M1 coefficient to 0.748 ± 0.141, suggesting an enhancement of PINK-1/PARKIN colocalization compared with control. A comparable effect was observed for biochanin A (5 μM), which produced the highest mean M1 value (0.780 ± 0.086), indicating the strongest stimulation of PINK-1 recruitment to PARKIN-positive mitochondria among the tested compounds. Curcumin (20 μM) resulted in an M1 value of 0.658 ± 0.081, remaining close to control levels, whereas CAPE (25 μM) produced a moderate increase (0.718 ± 0.053). In contrast, CurE (2.5 μM) reduced the M1 coefficient to 0.501 ± 0.119, the lowest colocalization level among treated WEHI-164 cells, suggesting attenuation of the PINK-1/PARKIN interaction under this condition. Overall, most compounds maintained or enhanced PINK-1/PARKIN colocalization in WEHI-164 cells, with biochanin A exerting the most pronounced effect. In L6 myoblasts, the basal M1 coefficient in control cells was markedly lower (0.350 ± 0.072) than in WEHI-164 cells, indicating a reduced constitutive colocalization of PINK-1 with PARKIN in non-tumor muscle cells. Exposure to berberine (50 μM) led to a slight decrease in M1 (0.253 ± 0.038). A more pronounced reduction was observed following treatment with curcumin (20 μM) (0.159 ± 0.047) and CurE (2.5 μM) (0.206 ± 0.071), suggesting diminished recruitment of PINK-1 to PARKIN-positive structures. In contrast, biochanin A (5 μM) significantly increased the M1 coefficient to 0.443 ± 0.036, the highest among the L6 experimental groups and exceeding the control level. CAPE (25 μM) resulted in a moderate decrease (0.265 ± 0.027) compared with the control. Thus, in L6 cells, most tested compounds reduced PINK-1/PARKIN colocalization, except for biochanin A, which enhanced their interaction.

The reciprocal Manders’ coefficient (M2), representing the fraction of the PARKIN signal colocalizing with PINK-1, was also analyzed (Figure 6b). In control WEHI-164 cells, the mean M2 value was 0.600 ± 0.218, indicating that approximately 60% of the total PARKIN signal colocalized with PINK-1 under basal conditions. Treatment with berberine (50 μM) increased the M2 coefficient to 0.847 ± 0.075, reflecting enhanced recruitment of PARKIN to PINK-1-positive mitochondria. A similar increase was observed following exposure to curcumin (20 μM), resulting in a mean M2 value of 0.907 ± 0.067. CAPE (25 μM) and CurE (2.5 μM) produced comparably elevated M2 values (0.884 ± 0.049 and 0.918 ± 0.053, respectively), with CurE yielding the highest overall level of colocalization in WEHI-164 cells. In contrast, biochanin A (5 μM) induced a more moderate increase, with an M2 coefficient of 0.755 ± 0.025, remaining above control but below that of the other tested compounds. Collectively, all analyzed compounds increased the proportion of PARKIN signal overlapping with PINK-1 in WEHI-164 cells, suggesting enhanced engagement of the PINK-1/PARKIN axis in this tumor-derived cell line.

In L6 myoblasts, the basal M2 coefficient in control cells was markedly lower (0.376 ± 0.102) compared with WEHI-164 cells, indicating reduced constitutive PARKIN localization to PINK-1-positive structures. Exposure to berberine (50 μM) moderately increased M2 to 0.519 ± 0.038, while CAPE (25 μM) produced a comparable value (0.433 ± 0.021). Curcumin (20 μM) decreased the M2 coefficient to 0.285 ± 0.069, representing the lowest colocalization level among L6 treatment groups. A pronounced increase was observed following biochanin A (5 μM) treatment, with M2 reaching 0.644 ± 0.052, indicating enhanced PARKIN recruitment in L6 cells under this condition. Notably, CurE (2.5 μM) resulted in the highest M2 value in L6 myoblasts (0.635 ± 0.079), suggesting substantial stimulation of PARKIN/PINK-1 colocalization. Thus, in L6 cells, the response was more heterogeneous: some compounds (biochanin A and CurE) markedly increased M2, whereas curcumin reduced it below basal levels.

Overall, the quantitative colocalization analysis revealed compound-dependent and cell line-dependent differences in both M1 and M2 Manders’ coefficients, with statistically significant effects observed relative to untreated control cells.

### 2.6. SA-β-Galactosidase Activity in L6 and WEHI-164 Cells After Exposure to Cytotoxic Compounds

After 48 h of incubation with the tested compounds, cellular senescence was assessed by measuring SA-β-gal enzyme activity.

**Figure 7:**
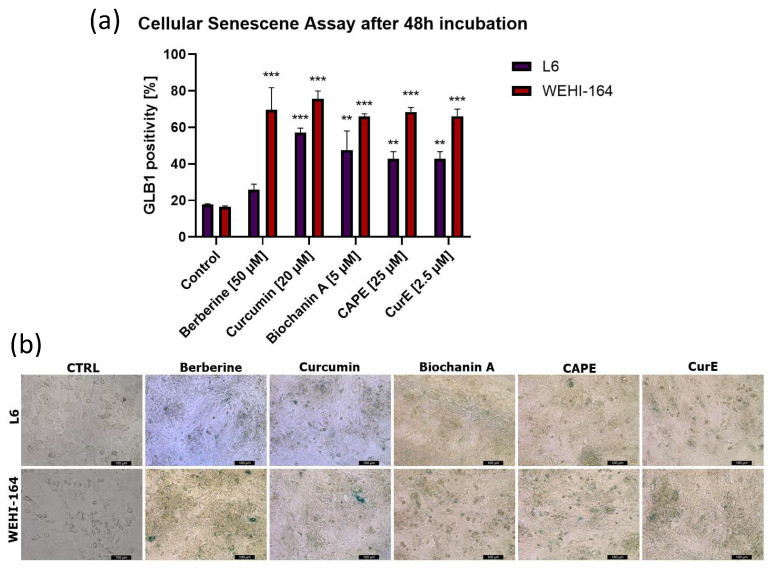
SA-β-gal enzyme activity presence in normal and cancerous cells after 48 h exposure to the tested compounds. Error bars represent ± SD for N = 3. Statistical significance relative to the control cells of the respective line was determined using one-way analysis of variance (ANOVA), where ** indicates *p* < 0.001, and *** *p* < 0.005 (**a**). Presentation of the progression of senescence in normal L6 and cancerous WEHI-164 cells after 48 h exposure to berberine (50 μM), curcumin (20 μM), biochanin A (5 μM), CAPE (25 μM), and CurE (2.5 μM), visualized using an Olympus BX51 light microscope (Shinjuku, Tokyo, Japan). Senescent cells are visualized by blue-green staining following SA-β-gal detection. Scale bar—100 µm (**b**).

The percentage of SA-β-gal-positive cells in the untreated L6 control group was 17.83 ± 0.40%. Treatment with berberine (50 μM) resulted in a modest increase to 25.84 ± 3.08%, which was not statistically significant (Figure 7a). In contrast, curcumin (20 μM) markedly elevated the proportion of senescent cells to 57.11 ± 2.51% (*** *p* < 0.001 vs. control), corresponding to an approximately 3.2-fold increase compared with untreated cells. Biochanin A (5 μM) increased SA-β-gal positivity to 47.64 ± 10.44% (** *p* < 0.01), representing a 2.7-fold elevation relative to control. CAPE (25 μM) and CurE (2.5 μM) induced comparable effects, increasing the percentage of senescent cells to 42.78 ± 3.91% and 42.79 ± 3.90%, respectively (both ** *p* < 0.01), corresponding to approximately 2.4-fold increases. Overall, curcumin exerted the strongest pro-senescent effect in L6 cells, followed by biochanin A, CAPE, and CurE, whereas berberine demonstrated only a minor effect.

In WEHI-164 cells, the baseline percentage of SA-β-gal-positive cells was 16.50 ± 0.55%, comparable to L6 control values. Berberine treatment markedly increased senescence to 69.68 ± 12.10% (*** *p* < 0.001), representing more than a fourfold increase relative to control. Curcumin induced the highest level of senescence (75.56 ± 4.39%, *** *p* < 0.001), corresponding to a 4.6-fold elevation. Biochanin A increased SA-β-gal positivity to 66.02 ± 1.42% (*** *p* < 0.001), while CAPE and CurE resulted in 68.45 ± 2.53% and 66.11 ± 3.93%, respectively (both *** *p* < 0.001). Thus, all tested compounds strongly and significantly induced a senescent phenotype in WEHI-164 cells, with curcumin showing the most pronounced effect.

Although baseline SA-β-gal activity was comparable between L6 (17.83%) and WEHI-164 (16.5%) control cells, the magnitude of treatment-induced senescence differed substantially between the two cell lines. WEHI-164 cells exhibited a markedly stronger response to all compounds. Most notably, berberine induced only a slight increase in L6 cells (25.84%) but triggered a robust rise to 69.68% in WEHI-164 cells. Similarly, biochanin A, CAPE, and CurE produced moderate increases in L6 cells (approximately 45%), whereas in WEHI-164 cells, senescence consistently exceeded 66%. Curcumin was the most potent inducer in both models; however, the absolute level of senescence remained higher in WEHI-164 (75.56%) than in L6 cells (57.11%). Collectively, these findings indicate that WEHI-164 tumor cells are substantially more susceptible to treatment-induced senescence than normal L6 myoblasts, suggesting a differential cellular response to NF-κB-modulating compounds. The Figures below show photos of control and cancer cells after 48 h of exposure to the analyzed substances (Figure 7b). Senescent cells are visualized by blue-green staining following SA-β-gal detection.

### 2.7. Analysis of Rel-α, Rel-β, NF-κB1, NF-κB2, and PFKFB3 Expression in WEHI-164 Cells After Exposure to Cytotoxic Compounds

**Figure 8:**
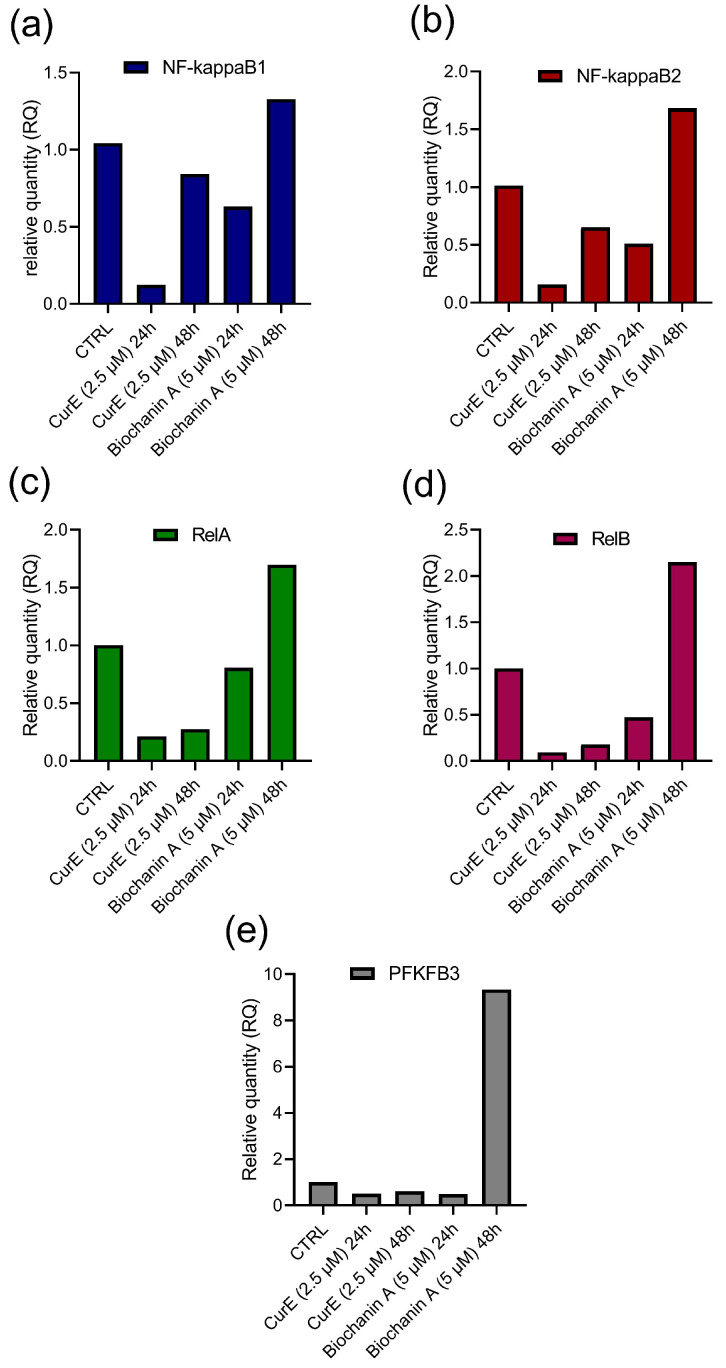
Validation of NF-κB1 (**a**), NF-κB2 (**b**), Relα (**c**), Relβ (**d**), PFKFB3 (**e**) gene expression in WEHI-164 cancer cells after 24- and 48 h exposure to CurE (2.5 µM) and biochanin A (5 µM) using real-time PCR.

To evaluate the effects of biochanin A and CurE on NF-κB pathway activity and glycolytic metabolism, the expression of five genes—NF-κB1, NF-κB2, RelA, RelB, and PFKFB3—was analyzed in WEHI-164 cancer cells after 24 and 48 h of incubation with the tested compounds. mRNA expression was quantified by qPCR, and the results are presented as relative transcript levels (RQ) compared to the control sample. Analysis of NF-κB1 expression revealed significant changes in response to the applied compounds. The lowest transcript levels were observed in cells treated with CurE for 24 h (~0.2RQ), indicating a pronounced inhibitory effect. However, after 48 h, NF-κB1 expression increased (~0.8 RQ), indicating partial recovery toward the control level. In the case of biochanin A, a variable effect was observed—after 24 h, NF-κB1 expression was reduced (~0.6 RQ), whereas after 48 h it increased significantly (~1.4 RQ), suggesting a rebound effect (Figure 8).

In the case of NF-κB2, a similar pattern was observed. CurE markedly reduced gene expression after 24 h (~0.2 RQ), whereas after 48 h transcript levels increased (~0.6 RQ), indicating partial recovery toward the control level. Biochanin A also induced a time-dependent effect: expression decreased after 24 h (~0.5 RQ) but increased above the control level after 48 h (~1.6 RQ), suggesting a compensatory response.

A different pattern was observed for the Rel-α gene. CurE markedly reduced its expression at both time points (~0.2 RQ after 24 h and ~0.3 RQ after 48 h), indicating sustained suppression without recovery toward the control level. Biochanin A induced a biphasic response. Rel-α expression decreased after 24 h (~0.8 RQ) and subsequently increased above the control level after 48 h (~1.7 RQ), indicating a time-dependent rebound effect.

Rel-β expression showed pronounced time-dependent changes. CurE caused a significant reduction in transcript levels (~0.1 RQ after 24 h and ~0.2 RQ after 48 h of incubation), indicating sustained suppression. In contrast, biochanin A initially reduced expression (~0.45 RQ after 24 h), followed by a strong increase after 48 h (~2.1 RQ), exceeding the control level. The most pronounced changes were observed for the PFKFB3 gene, which encodes a key glycolytic regulatory enzyme. Expression levels after 24 h of incubation with CurE and biochanin A remained low (~0.5RQ). After 48 h of exposure to biochanin A, a sharp increase in expression was observed, reaching values close to 9 RQ, whereas CurE did not induce a comparable effect.

Overall, CurE reduced the expression of NF-κB-related genes, with partial recovery observed after 48 h, although transcript levels remained below the control. In contrast, biochanin A induced a time-dependent biphasic response, characterized by an initial decrease followed by increased expression after 48 h, accompanied by a marked upregulation of the glycolytic regulator *PFKFB3*. This pronounced increase in *PFKFB3* expression may reflect altered transcriptional regulation of glycolysis-associated genes. Taken together, CurE exerted a sustained inhibitory effect on NF-κB-related gene expression, whereas biochanin A, particularly after prolonged exposure, was associated with increased expression of NF-κB pathway components and *PFKFB3*. These findings indicate that the two compounds differentially modulate transcriptional responses related to inflammatory signaling and cellular metabolism in murine sarcoma cells.

### 2.8. Toxicity Testing of the Analyzed Compounds in Galleria mellonella Larvae

**Figure 9:**
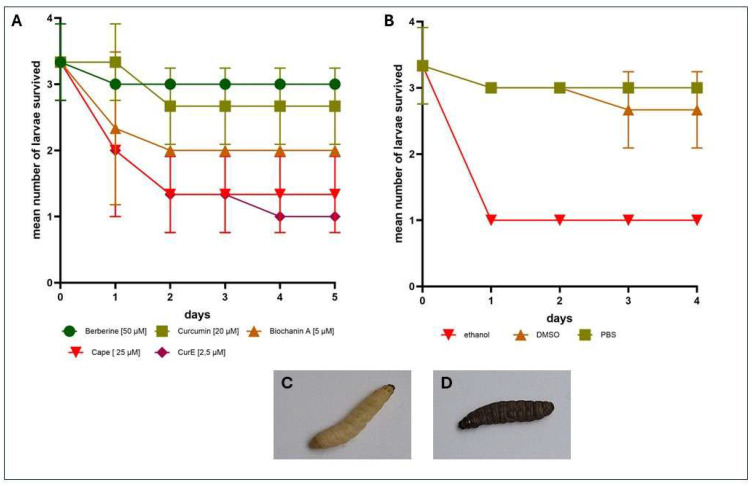
Survival of *Galleria mellonella* larvae after treatment with the analyzed substances (**A**) and control solutions (**B**). (**C**) healthy *Galleria mellonella* larvae; (**D**) *Galleria mellonella* larvae after treatment with 70% ethanol.

The toxicity of the natural compounds was assessed in *Galleria mellonella* larvae (Figure 9). Each group initially contained 10 larvae. PBS served as the handling (negative) control, and 70% ethanol as the utility (positive) control; an additional DMSO vehicle control was included to account for the solvent used to prepare the compound stocks.

In the 70% ethanol group, the number of living larvae decreased to 3 after 24 h, and complete mortality was observed the following day. In contrast, larvae injected with PBS or DMSO showed high survival throughout the experiment (9/10 and 8/10 larvae alive at day 5, respectively). Among the tested compounds, berberine [50 μM] was best tolerated, with 9 larvae alive throughout the observation period. Curcumin [20 μM] caused no mortality during the first day; survival gradually decreased to 8 larvae on days 2–4 and to 7 larvae on day 5. CAPE [25 μM] resulted in 40% survival at the end of the experiment. CurE [2.5 μM] reduced survival to 7 larvae on day 1, to 4 larvae on days 2–3, and to 3 larvae on day 5. The most pronounced toxic effect was observed for biochanin A [5 μM], in which the number of surviving larvae dropped to 2 within the first 24 h and remained at this level until the end of the observation period.

Survival data were analyzed using Kaplan–Meier survival curves and the log-rank test. The analysis revealed significant overall differences among the tested compounds (χ^2^ = 10.81, df = 4, *p* = 0.0287) and among all experimental groups, including the vehicle controls (χ^2^ = 21.89, df = 7, *p* = 0.0027). Pairwise comparisons indicated that berberine and curcumin did not differ significantly from the PBS or DMSO controls, whereas CAPE and CurE showed a significantly stronger survival-reducing effect. Biochanin A produced the most pronounced mortality across all comparisons. The *G. mellonella* assay was interpreted as a preliminary acute toxicity screen only, and not as evidence of systemic safety or as a substitute for mammalian pharmacokinetic or chronic toxicity studies. Within these limitations, the assay successfully differentiated compounds with low and high acute toxic potential. The lack of significant larval toxicity for berberine and curcumin supports their prioritization for follow-up evaluation in mammalian models, while the pronounced toxicity of biochanin A and CurE indicates that caution is warranted regarding their further biological application.

## 3. Discussion

The present study suggests that natural compounds historically associated with NF-κB modulation cannot be treated as a mechanistically uniform group in fibrosarcoma. Although all five compounds affected cell viability and stress-related endpoints, they differed markedly in the extent to which they disrupted mitochondrial homeostasis, engaged PINK1/PARKIN-associated mitochondrial quality control, induced senescence, and preserved an acceptable safety window. Relative to earlier work in the same WEHI-164/L6 experimental setting, the current dataset broadens the interpretation from simple cytotoxicity to an integrated view of bioenergetics, mitophagy, senescence, NF-κB pathway-related transcriptional changes, and preliminary in vivo-like toxicity [38]. Taken together, the results support the concept that sarcoma cells are more vulnerable than normal myogenic cells to combined NF-κB- and mitochondria-directed stress, but they also indicate that this vulnerability is highly compound-specific and does not always translate into an advantageous therapeutic index in a whole-organism screening model.

An important first observation concerns the distinction between early apparent selectivity and later integrated stress sensitivity. Based on 24 h IC_50_ values, curcumin and CAPE were the most clearly tumor-selective compounds, whereas biochanin A, berberine, and especially CurE appeared relatively more toxic to L6 cells. However, when later endpoints were considered, including 48 h ATP depletion and senescence, WEHI-164 cells proved consistently more susceptible than L6 cells. This discrepancy suggests that short-term viability measurements do not fully capture delayed mitochondrial and transcriptional changes. In practical terms, curcumin and CAPE appear to combine the most convincing early selectivity with sustained antitumor activity, whereas CurE may be underestimated if judged only by the 24 h IC_50_ dataset. Conversely, compounds that appear strongly cytotoxic in vitro but lack clear tumor selectivity at early time points should be interpreted cautiously, particularly when their effects on normal myogenic bioenergetics are substantial.

The mitochondrial data are consistent with the stronger response of WEHI-164 cells. In L6 myoblasts, all compounds reduced JC-1 polarization, with CurE and berberine causing the strongest depolarization. In WEHI-164 cells, berberine and CAPE induced profound depolarization, whereas curcumin and CurE caused a more moderate fall in membrane potential, and biochanin A even increased the JC-1 red/green ratio. Importantly, however, ATP depletion after 48 h was markedly greater in WEHI-164 than in L6 cells for all compounds, indicating that mitochondrial membrane potential and terminal bioenergetic competence were not perfectly coupled in this model. This is biologically plausible. Berberine is known to inhibit mitochondrial respiratory complex I and to activate AMPK through energetic stress, while in cancer cells, it can also reduce mitochondrial membrane potential and ATP content [39,40]. CAPE has likewise been shown to provoke mitochondrial depolarization while suppressing NF-κB-dependent signaling in carcinoma models [41]. The present results, therefore, fit well with a model in which sarcoma cells have lower tolerance to sustained energetic stress: even when the fall in ΔΨm is not maximal, downstream ATP collapse may still be profound because tumor cells are less capable of restoring energetic balance under continued NF-κB- and mitochondria-directed challenge.

The mitophagy findings add a further layer to this interpretation. Basal PINK1/PARKIN colocalization was higher in WEHI-164 than in L6 cells, consistent with greater constitutive mitochondrial stress in tumor cells. Following treatment, berberine and CurE markedly stimulated mitophagy in both cell types, but CurE and CAPE produced especially strong effects in WEHI-164 cells. At the same time, all compounds increased the M2 coefficient in WEHI-164 cells, indicating enhanced PARKIN localization to PINK1-positive structures. As mitophagy was assessed by PINK1/PARKIN colocalization and a mitophagy dye, the present data refer to PINK1/PARKIN-associated mitophagy and not to autophagic flux in general (LC3B-II/I ratio and p62 turnover were not measured). These findings suggest that tumor cells were actively engaging the PINK1/PARKIN axis in response to the tested agents. The functional meaning of this response is likely context-dependent. In some cancer systems, PINK1/PARKIN-dependent mitophagy contributes directly to antitumor activity by promoting the clearance of damaged mitochondria, a process incompatible with tumor survival [42]. In others, mitophagy is protective and can blunt drug lethality by limiting the accumulation of mitochondrial damage [43]. In the present study, the combination of strong mitophagy, major ATP depletion, and reduced viability—particularly for CurE, berberine, and CAPE—suggests that mitophagy was insufficient to restore homeostasis and may instead reflect a stressed, ultimately overwhelmed adaptive response. Thus, activation of the PINK1/PARKIN axis in WEHI-164 cells should not automatically be interpreted as cytoprotection; in this setting, it may mark a threshold beyond which mitochondrial damage becomes incompatible with tumor-cell survival.

Biochanin A deserves particular attention because it produced the most paradoxical phenotype. In WEHI-164 cells, it induced strong cytotoxicity, marked ATP depletion, and robust senescence, yet it did not behave like a straightforward mitochondrial poison. Instead of depolarizing mitochondria, biochanin A increased mitochondrial membrane polarization and only mildly elevated mitophagy in WEHI-164 cells, while strongly increasing the M1 coefficient and, later, producing a clear rebound in transcripts of NF-κB pathway components together with an approximately nine-fold increase in PFKFB3 mRNA levels. This pattern contrasts with the more canonical pro-apoptotic and NF-κB-suppressive behavior reported for biochanin A in other tumor models, where the compound enhanced apoptosis and reduced NF-κB signaling [44,45]. One plausible interpretation is that biochanin A triggers an early adaptive rather than terminal mitochondrial response in WEHI-164 cells, with transient preservation or hyperpolarization of mitochondrial function followed by late bioenergetic failure. The sharp increase in PFKFB3 mRNA after 48 h is a striking transcriptional change consistent with, but not direct evidence of, glycolytic compensation. Because PFKFB3 is a key regulator of glycolytic flux and is implicated in tumor-associated metabolic adaptation [46,47], this late transcriptional response is intriguing; however, without loss-of-function or pharmacological inhibition of PFKFB3, we cannot determine whether the response protects WEHI-164 cells from biochanin A or merely accompanies its cytotoxicity. This is therefore presented as a descriptive, hypothesis-generating observation rather than a causal mechanism. From a translational perspective, the combination of mechanistically unpredictable cellular effects and noticeable toxicity in the larval model makes biochanin A less attractive than its in vitro potency alone might suggest.

CurE showed a substantially different profile and emerged as one of the most phenotypically coherent compounds in the study. It caused strong induction of PINK1/PARKIN-associated mitophagy in WEHI-164 cells, pronounced ATP depletion, marked senescence, and sustained down-regulation of NF-κB1, NF-κB2, Relα, and Relβ transcripts, while not provoking the late PFKFB3 rebound seen with biochanin A. This coordinated transcriptional pattern is dependent on the possibility that CurE may suppress both inflammatory-survival signaling and glycolysis-associated adaptation. Such an interpretation is consistent with the broader cucurbitacin literature, in which cucurbitacins promote cell-cycle arrest, mitochondrial apoptosis, and, in some settings, ROS-linked autophagy [48,49,50]. The apparently discordant colocalization data—reduced M1 but very high M2 in WEHI-164 cells—do not necessarily contradict pathway activation; rather, they may indicate altered stoichiometry or flux within the PINK1/PARKIN system during active mitochondrial turnover. Nonetheless, CurE also exhibited a narrow safety margin: its in vitro effects on L6 mitochondria were strong. In the Kaplan–Meier analysis of the *Galleria mellonella* survival data, CurE produced a stronger survival-reducing effect than curcumin or berberine. CurE should therefore be regarded as a potent but pharmacologically demanding lead whose therapeutic promise will depend on dose optimization, delivery strategy, and confirmation in mammalian models.

Another major finding is the strong induction of senescence, particularly in WEHI-164 cells. All five compounds markedly increased SA-β-gal positivity in the tumor line, and curcumin produced the strongest effect. This is noteworthy because therapy-induced senescence can constitute a meaningful antitumor endpoint by imposing durable proliferative arrest even when cell death is incomplete [51]. At the same time, senescence is biologically ambivalent, as its long-term consequences depend on the composition of the senescence-associated secretory phenotype (SASP), which is in part regulated by NF-κB signaling [51]. In this context, the transcriptional divergence between CurE and biochanin A is particularly informative. CurE maintained down-regulation of NF-κB pathway transcripts, whereas biochanin A displayed late overexpression of these transcripts. Although SASP markers were not measured directly, one may cautiously speculate that CurE-induced senescence could be less inflammation-promoting than the biochanin A-associated state. More broadly, the senescence results are consistent with the mitochondrial data, as persistent mitochondrial dysfunction is a recognized driver of senescence-associated cell-cycle arrest and stress signaling [52]. Curcumin is also particularly interesting here: its preferential cytotoxicity towards WEHI-164 cells, combined with the strongest senescence induction, is consistent with the proposed role of curcumin in attenuating NF-κB-dependent survival programs in fibrosarcoma-like cells, thereby favoring strong growth arrest and loss of viability [53].

The *G. mellonella* assay substantially strengthens the translational interpretation of the in vitro findings because it shows that efficacy-related cellular stress does not necessarily predict acceptable organismal tolerability. Berberine and curcumin were the best-performing compounds in the larval model, with minimal mortality over the observation period. CAPE showed intermediate toxicity, whereas CurE and especially biochanin A were clearly less well tolerated. These data are important because they reveal the practical attractiveness of the compounds. Biochanin A, despite its potent in vitro activity, becomes difficult to prioritize because its cellular effects are mechanistically inconsistent and its whole-organism toxicity is high. CurE remains interesting mechanistically but raises safety concerns. By contrast, curcumin—and to a lesser extent berberine—combine strong antitumor activity with the most favorable preliminary safety profile. This use of *G. mellonella* is justified, as the model has been shown to be useful for acute toxicity testing and to display meaningful concordance with mammalian toxicity trends in some xenobiotic studies [54,55]. Accordingly, the larval assay in the present work should be viewed as a valuable validation tool rather than a substitute for mammalian pharmacokinetic, biodistribution, or chronic toxicity studies.

Several limitations should be considered when interpreting these findings. First, the study compares two rodent cell lines of different tissue and species backgrounds rather than an isogenic tumor/normal pair. Therefore, part of the differential response may reflect baseline biological differences unrelated to malignant transformation. Secondly, the transcriptional analysis was restricted to WEHI-164 cells and to only two compounds; the mechanistic ranking cannot yet be generalized across the full compound panel. Thirdly, NF-κB activity was inferred from mRNA expression of pathway components rather than from protein phosphorylation, nuclear translocation, or DNA-binding assays. Fourthly, some mitochondrial and mitophagy measurements were performed with limited replicate numbers, so the direction of effect is convincing, but the quantitative precision remains modest. Finally, the Galleria assay does not address mammalian pharmacokinetics, tissue distribution, or chronic toxicity. Future work should therefore include human sarcoma models, direct analysis of NF-κB activation state, Seahorse-based bioenergetic profiling, glycolytic flux measurements, and combination studies with standard sarcoma therapeutics. In particular, the biochanin A-associated PFKFB3 rebound suggests that metabolic co-targeting may be a rational next step [46,47].

Overall, the data support a model in which natural NF-κB modulators exert anticancer activity in murine fibrosarcoma primarily by destabilizing mitochondrial and metabolic homeostasis, with tumor cells displaying greater downstream vulnerability than normal myogenic cells. Among the tested agents, curcumin appears to offer the most balanced profile, combining preferential cytotoxicity, strong senescence induction, and low larval toxicity. CAPE also emerges as a promising candidate because of its tumor-selective cytotoxicity and pronounced mitochondrial effects, although its safety margin appears narrower than that of curcumin. Berberine shows robust bioenergetic activity and good tolerability in the larval assay, but its effects on normal muscle mitochondria warrant caution. CurE is mechanistically compelling but limited by safety concerns, whereas biochanin A appears least suitable for further development in its current form because its delayed NF-κB/PFKFB3 rebound and high organismal toxicity undermine its translational value. These distinctions are important because they suggest that future development should prioritize not merely NF-κB inhibition per se, but compound-specific patterns of mitochondrial disruption, metabolic adaptation, and tolerability.

## 4. Materials and Methods

### 4.1. Cell Cultures

Two cell lines were used in in vitro studies: WEHI-164-fibrosarcoma cells isolated from mice and L6—myogenic cell line isolated from skeletal muscles of rats (ATCC^®^, LGC Standards, Teddington, UK). The WEHI-164 cells were grown in RPMI culture medium supplemented with 10% fetal bovine serum (FBS, HyClone, Logan, UT, USA) and 1% penicillin/streptomycin (Sigma-Aldrich, Merck-Millipore, Poznan, Poland). The L6 cells were grown in DMEM culture medium (Dulbecco’s Modified Eagle’s Medium, Sigma-Aldrich, Merck-Millipore, Poznan, Poland) with 10% fetal bovine serum (FBS, HyClone, Logan, UT, USA), 1% penicillin/streptomycin (Sigma-Aldrich, Merck-Millipore, Poznan, Poland), and 1% L–glutamine (ThermoFisher, Alab, Warsaw, Poland). Cell culture of both cell line types was carried out at 37 °C and 5% CO_2_. Both cell lines were negative for mycoplasma (MycoBlue Mycoplasma Detector, Vazyme Biotech, dist. Gentaur, Sopot, Poland. Cell passages were performed 2–3 times a week when confluency was 80–90%. Cells were then removed from the flasks by trypsinization (0.25% trypsin and 0.02% EDTA; IITD, Wroclaw, Poland) and washed with DPBS buffer (Sigma-Aldrich, Merck-Millipore, Poznan, Poland).

### 4.2. Preparation of Drug Solutions

CurE (number CAS 18444-66-1), biochanin A (number CAS 207-744-7), CAPE (number CAS 104594-70-9), curcumin (number CAS 458-37-7), and berberine (number CAS 633-65-8) were obtained from Sigma-Aldrich (Merck-Millipore, Poznan, Poland). Stock solutions were prepared in dimethyl sulfoxide (DMSO) according to the manufacturer’s recommendations. Immediately prior to the experiments, the compounds were diluted in culture medium (DMEM or RPMI-1640, depending on the cell line) to obtain the desired final concentrations. CurE was tested at concentrations of 0.5, 1, 2, 2.5, 3, and 5 μM. Biochanin A was used at 0.5, 2, 5, 10, 20, and 30 μM. CAPE was used at 1, 5, 10, 15, 25, and 50 μM. Curcumin and berberine were tested at 5, 10, 15, 20, 25, and 50 μM. Control cells were treated with culture medium containing the corresponding DMSO concentration without test compounds.

### 4.3. Measurement of Mitochondrial Membrane Potential

WEHI-164 and L6 cells were seeded onto microscopic glass coverslips placed in 6-well culture plates (Sarstedt, Nümbrecht, Germany) and incubated overnight to allow cell adhesion. The following day, the cells were treated for 24 h with selected compounds at the following concentrations: biochanin A (5 μM), CAPE (25 μM), CurE (2.5 μM), curcumin (20 μM), or berberine (50 μM). Control cells were cultured under the same conditions without the addition of test compounds. Mitochondrial membrane potential was assessed using the fluorescent dye JC-1 (ThermoFisher Scientific, Waltham, MA, USA). The dye was diluted 1:100 in complete culture medium (DMEM for L6 cells and RPMI-1640 for WEHI-164 cells). A volume of 350 μL of the JC-1 fluorescent solution was added to each sample, followed by incubation for 20 min at 37 °C in a humidified atmosphere containing 5% CO_2_. Observations were performed using an Olympus Fluoview FV1000 confocal laser scanning microscope (Olympus, Tokyo, Japan). Two biological replicates and three technical replicates were performed; at least 300 cells were analyzed per condition. The images were analyzed with ImageJ software (LOCI, University of Wisconsin). The same JC-1 quantification approach—red/green fluorescence ratio normalized to the untreated control group of each condition—has been previously applied and published in Cells [56]. The ratio of red-to-green fluorescence intensity was used as an indicator of mitochondrial membrane potential. The experiment was performed in duplicate.

### 4.4. Measurement of Mitophagy Intensity

WEHI-164 and L6 cells were seeded onto microscopic glass coverslips placed in 6-well culture plates (Sarstedt, Germany) and incubated overnight to allow adhesion. Subsequently, the cells were washed twice with HBSS (Hank’s Balanced Salt Solution). Mitophagy was assessed using the Mitophagy Detection Kit (Dojindo Laboratories, Kumamoto, Japan) according to the manufacturer’s instructions. Briefly, the Mitophagy Dye was diluted in an appropriate serum-free culture medium to a final concentration of 0.1 μM, and 350 μL of the staining solution was added to each well. The cells were incubated for 20 min at 37 °C in a humidified atmosphere containing 5% CO_2_ and then washed with HBSS. Subsequently, they were incubated for 24 h with one of the selected cytotoxic compounds: biochanin A (5 μM), CAPE (25 μM), CurE (2.5 μM), curcumin (20 μM), or berberine (50 μM). Control cells were cultured under the same conditions without test compounds. The Mitophagy Dye is retained within intact mitochondria, exhibiting weak fluorescence. Upon mitochondrial damage and fusion with lysosomes, a marked increase in dye fluorescence is observed. Observations were performed using an Olympus Fluoview FV1000 confocal laser scanning microscope (Olympus, Japan), and the images were analyzed with ImageJ software v.1.53 (LOCI, University of Wisconsin). At least 50 cells were analyzed per condition. The experiment was performed in duplicate.

### 4.5. MTT Viability Assay

WEHI-164 and L6 cells were seeded onto 96-well plates at a density of 1 × 10^4^ cells per well and incubated with berberine, curcumin, biochanin A, CAPE, and CurE at specific concentrations and time intervals. Subsequently, the culture medium was removed from each well, and 100 μL of reagent [3-(4,5-dimethylthiazol-2-yl)-2,5-diphenyltetrazolium bromide] (Sigma-Aldrich, dist. Merck-Millipore, Poznan, Poland) was added to each well. The cells were then incubated for 2 h at 37 °C. After incubation, acidified isopropanol (100 μL, 0.04 M HCl in 99.9% isopropanol) was added to dissolve the resulting formazan crystals. Dissolution was facilitated by pipette mixing. Absorbance was measured at a wavelength of 570 nm using a GloMax^®^ Discover microplate reader (Promega, Madison, WI, USA). IC_50_ values were calculated using the Quest Graph™ IC50 Calculator (AAT Bioquest, Inc., Sunnyvale, CA, USA) based on a nonlinear regression model. All experiments were performed in five replicates. The results were expressed as the percentage of viable cells relative to the untreated control.

### 4.6. Cell Viability Test—PrestoBlue™

WEHI-164 and L6 cells were seeded onto black, clear-bottom 96-well plates at a density of 1 × 10^4^ cells per well and incubated with berberine, curcumin, biochanin A, CAPE, and CurE at specified concentrations and time intervals. Subsequently, the PrestoBlue™ reagent was diluted in culture medium at a 1:10 ratio according to the manufacturer’s instructions and added. After a 10 min incubation at 37 °C in a humidified atmosphere containing 5% CO_2_, fluorescence was measured at an excitation/emission wavelength of 590–615 nm using a GloMax Discover plate reader (Promega, Madison, WI, USA). The experiments were performed in five replicates. The obtained results were analyzed according to the following formula:

$$\%\;\mathrm{cell}\;\mathrm{viability}=\frac{mean\;fluorescence\;value\;of\;treated\;cells}{mean\;fluorescence\;value\;of\;control\;cells}\times 100\%$$

### 4.7. Analysis of PARKIN and PINK-1 Using Confocal Microscopy

WEHI-164 and L6 cells were seeded onto microscopic glass coverslips placed in 6-well culture plates (Sarstedt, Germany) and incubated overnight to allow adhesion. Subsequently, the cells were incubated for 24 h with one of the selected cytotoxic compounds: biochanin A (5 μM), CAPE (25 μM), CurE (2.5 μM), curcumin (20 μM), or berberine (50 μM). Control cells were cultured under the same conditions without test compounds. After incubation, the cells were fixed with a 4% paraformaldehyde solution in phosphate-buffered saline (PBS) and subsequently permeabilized with 0.5% Triton X-100 in PBS for 5 min. To block nonspecific binding sites, a 1% bovine serum albumin (BSA) solution in PBS was applied, and the cells were incubated at RT (room temperature) for 45 min. All washing steps were performed using PBS. The following primary antibody was used in the study: PINK-1 (rabbit, PA5-23072), diluted 1:250 in PBS. Mouse PARKIN (SC32282) was detected using a secondary antibody conjugated to Alexa Fluor 647 at a dilution of 1:250 in PBS. Incubation with primary antibodies was performed for 2 h, followed by incubation with the secondary antibody conjugated to Alexa Fluor^®^ 488 (A11001) (diluted 1:200) for 1 h. In both cases, incubation was carried out at 37 °C in a humidified atmosphere containing 5% CO_2_. After the final wash with PBS, Fluoroshield™ mounting medium with DAPI (Sigma-Aldrich, St. Louis, MO, USA) was applied to counterstain cell nuclei and mount the samples onto glass slides. For each experimental condition, seven independent microscopic images were acquired and subsequently analyzed for colocalization using ImageJ software v.1.53 (LOCI, University of Wisconsin). Manders’ coefficients (M1 and M2) were calculated to determine the rate of total PINK1 signal colocalizing with PARKIN (M1) and the percentage of total PARKIN signal colocalizing with PINK1 (M2). Manders’ coefficient ranges from 0 to 1, where 0 indicates no colocalization and 1 indicates complete colocalization. Observations were performed using an Olympus FV1000 confocal microscope equipped with a Plan Apo 60×/1.35 NA oil-immersion objective in Sequential Scan mode.

### 4.8. Cellular Senescence Assay

A classical feature of the senescent phenotype is the induction of senescence-associated β-galactosidase (SA-β-gal) activity. SA-β-gal is expressed exclusively in senescent cells and is absent in quiescent or proliferating cells. WEHI-164 and L6 cells were seeded onto 6-well plates (Sarstedt, Germany) and incubated overnight to allow cell adhesion. Subsequently, the cells were treated for 48 h with one of the selected cytotoxic compounds: biochanin A (5 μM), CAPE (25 μM), CurE (2.5 μM), curcumin (20 μM), or berberine (50 μM). Untreated cells served as the control group. Cells were washed twice with PBS. The experiment was performed using the Cellular Senescence Assay (KAA002) purchased from Sigma-Aldrich (Merck-Millipore, Poznan, Poland). Cells were fixed by incubation with Fixing Solution at room temperature for 15 min, followed by two washes with PBS. The cells were then incubated in the dark for 4 h at 37 °C, in a CO_2_-free environment, using the SA-β-gal detection solution prepared according to the manufacturer’s instructions. Following incubation, cells were washed twice with PBS. Blue-green-stained cells were imaged using an Olympus BX51 light microscope (Shinjuku, Tokyo, Japan). Measurements were performed on at least 50 cells per condition. The experiment was conducted in three independent replicates.

### 4.9. ATP Level Measurement

WEHI-164 and L6 cells were seeded onto 6-well plates (Sarstedt, Germany) and incubated overnight to allow cell adhesion. Subsequently, the cells were treated for 48 h with one of the selected cytotoxic compounds: biochanin A (5 μM), CAPE (25 μM), CurE (2.5 μM), curcumin (20 μM), or berberine (50 μM). Untreated cells served as the control group. Cells were washed with PBS and then trypsinized. The cells were resuspended in PBS and centrifuged at 2000 rpm for 5 min. The pellet was incubated at room temperature for 10 min in lysis buffer (1% Triton X-100, 1 mM EDTA, 50 mM TRIS, pH 7.5). The cell suspension was transferred to Eppendorf tubes and heated at 99 °C for 90 s. Subsequently, the samples were centrifuged at 3000 rpm for 10 min at 4 °C and then cooled for 15 min at 4 °C. A reaction mixture was prepared by combining 500 µL of assay buffer (50 mM TRIS/HCl, 4 mM MgSO_4_, 30 mM K_2_SO_4_, and 1.7 mM EDTA, pH 7.4), 10 µL of the cell homogenate, and 50 µL of luciferase extract (FLE250) purchased from Sigma-Aldrich (Merck-Millipore, Poznań, Poland). Aliquots of 150 µL of the reaction mixture were transferred to wells of a 96-well luminescence plate. Luminescence was measured using a GloMax^®^ Discover microplate reader (Promega, Madison, WI, USA). The experiment was performed in three independent replicates.

### 4.10. Analysis of Rel-α, Rel-β, NF-κB1, NF-κB2, and PFKFB3 Expression in WEHI-164 Cells

WEHI-164 cells were cultured as described in Section 2.1. (Cell cultures). When approximately 80% confluence was reached, cells were treated with biochanin A (5 µM) or CurE (2.5 µM) for 24 and 48 h. Untreated cells served as the control group. Cells were collected from culture flasks, centrifuged at 500× *g* for 5 min, and the resulting cell pellets were stored at –20 °C for up to 1 week prior to further analyses. Total RNA was extracted using the GeneMATRIX Universal RNA Purification Kit (EURx, Gdansk, Poland, E3598-02) following the manufacturer’s protocol. Reverse transcription (RT) was performed to synthesize cDNA using 500 ng of isolated RNA, Random Hexamers (Thermo Fisher Scientific, Waltham, MA, USA, 8080127), and MultiScribe™ Reverse Transcriptase (Thermo Fisher Scientific, Waltham, MA, USA, 4311235). Subsequently, 3 µL of the threefold-diluted RT products were used for individual quantitative real-time PCR (qPCR) analyses with Fast Probe qPCR Master Mix (EURx, E0422-03) and gene-specific TaqMan probes: Mm00501346_m1 for RelA, Mm00485664_m1 for RelB, Mm00476361_m1 for NF-κB1, Mm00479807_m1 for NF-κB2, and Mm00504650_m1 for PFKFB3, with ACTB serving as the reference gene (all probes from Thermo Fisher Scientific, Waltham, MA, USA). All samples were analyzed in 96-well plates using the following thermal cycling conditions: initial denaturation at 95 °C for 3 min, followed by 40 cycles of denaturation at 95 °C for 10 s and annealing/extension at 60 °C for 30 s. Reactions were performed on a TOptical Real-Time PCR thermocycler (Biometra GmbH, Göttingen, Germany), and cycle threshold (Ct) values were obtained using qPCRsoft 4.1 software (Biometra GmbH, Göttingen, Germany). Relative quantification (RQ) of gene expression was calculated using the comparative Ct method (2^−ΔΔCt^), normalizing target gene expression to ACTB mRNA levels.

### 4.11. In Vivo Toxicity Test on Galleria mellonella Larvae

To assess the in vivo toxicity of the tested solutions, *Galleria mellonella* larvae (final larval stage, body mass: 200–250 mg) were used. Larvae were bred in standardized conditions at the Department of Translative Technologies. The larval population chosen for analysis was randomly divided into seven groups (n = 10 larvae per group). Each larva received 10 µL of the appropriate test solution, injected into the last left proleg using a Hamilton syringe (model 701RN, 10 µL) equipped with a 26S needle. Larvae were incubated in the dark at 37 °C, and their condition was monitored daily for 5 days. Assessment criteria included survival (recorded as alive or dead based on the absence of movement in response to touch) and the presence of melanization, classified as absent, partial, or full-body melanization. 70% ethanol was used as a positive toxicity control (utility control), whereas 0.9% sodium chloride (NaCl) served as a negative control (growth and handling control). All injections and assessments were performed under sterile conditions. Experiments were conducted in triplicate on separate days to ensure reproducibility of the results.

### 4.12. Statistical Analysis

Statistical analysis was performed in GraphPad Prism 8 (GraphPad Software Inc., San Diego, CA, USA). Data are expressed as mean ± SD. For cell-based assays, differences between treated groups and their matched untreated controls for each cell line were assessed using one-way ANOVA with Tukey’s post hoc test. For *Galleria mellonella* experiments, survival was analyzed by the Kaplan–Meier method, with pairwise comparisons between treatment groups and the 0.9% NaCl control performed using the log-rank (Mantel–Cox) test with Bonferroni correction for multiple comparisons. The significance notation used throughout the manuscript is: * *p* < 0.05; ** *p* < 0.01; *** *p* < 0.001; **** *p* < 0.0001.

## Figures and Tables

**Table 1:** IC_50_ values for the tested compounds determined after 24 h of treatment of L6 and WEHI-164 cells.

Cell Line/Compound	Biochanin A [µM]	CAPE [µM]	CurE [µM]	Berberine [µM]	Curcumin [µM]
L6	5.903	13.694	10.38	12.075	29.305
WEHI-164	6.395	7.018	66.476	27.043	14.049

**Table 2:** IC_50_ values for the tested compounds determined after 48 h of treatment of L6 and WEHI-164 cells.

Cell Line/Compound	Biochanin A [µM]	CAPE [µM]	CurE [µM]	Berberine [µM]	Curcumin [µM]
L6	2.846	5.211	1.944	5.807	17.427
WEHI-164	3.132	4.302	2.119	6.561	11.009

## Data Availability

The data presented in this study are available on request from the corresponding author.
